# A qualitative study of self-evaluation of junior doctor performance: is perceived ‘safeness’ a more useful metric than confidence and competence?

**DOI:** 10.1136/bmjopen-2015-008521

**Published:** 2015-11-04

**Authors:** Damian Roland, David Matheson, Timothy Coats, Graham Martin

**Affiliations:** 1 SAPPHIRE Group, Department of Health Sciences, University of Leicester, Leicester, UK; 2 Emergency Medicine Academic Group, University Hospitals of Leicester NHS Trust, Leicester, UK; 3 Carnegie Faculty, Leeds Beckett University, Leeds, UK

**Keywords:** MEDICAL EDUCATION & TRAINING, GENERAL MEDICINE (see Internal Medicine)

## Abstract

**Objectives:**

The terms confidence and competence have been poorly defined and are often misused by junior doctors. Given safe practice relies on healthcare professionals being aware of their own skill sets improving self-assessment of confidence and competence is important. The aim of this work was to explore junior doctors’ understanding of how they perceive their own performance in respect of managing feverish children in an emergency department.

**Setting:**

A children's emergency department in a tertiary hospital in the East Midlands, UK.

**Participants:**

22 Junior doctors volunteered to undertake focus groups via a meta-planning methodology over 2 years (14 participants in the first year and 8 in the second).

**Results:**

Although doctors were aware of the difference between confidence and competence they were not able to distinguish between them in practical terms. The feeling of being ‘safe’ emerged as a term in which there was a shared understanding compared to reported confidence and competence.

**Conclusions:**

A perception of ‘safeness’ is a concept that may aid self-evaluation and we present a matrix that might be used by supervisors and educators to examine this and its relationship with confidence and competence.

**Strengths and limitations of this study**

- This is the first evaluation of junior doctors’ understanding of the terms confidence and competence across two cohorts on doctors working in the same clinical environment.
- The study utilises a unique form of workshop, a meta-planning focus group, allowing participants to develop and analyse the ideas discussed by recording them on a whiteboard.
- The themes discussed were specific to the management of the feverish child so may not be applicable to other clinical presentations.

## Introduction

Self-assessment has long been a key component of medical education and revalidation or Continuing Medical Education of fully trained clinicians,^1^ ^2^ something Antonelli^3^ sees as ‘essential to the practice of medicine and self-directed life-long learning’. Self-assessment is a complex, potentially learnt skill, requiring individuals to have insights into their own limitations and competencies.^4^ This often takes place in feedback during work-place based assessments where the assessor enquires as to how they think the individual performed. In general terms when evaluating a training programme, researchers and managers are interested in whether participants have gained a greater belief in their abilities at carrying out a particular skill or knowledge set (*confidence*) and whether they are technically more proficient in putting them into practice (*competence*). However, there are variations on the definitions of competence and confidence in medical education.^5^ ^6^

Confidence and competence are associated but there is evidence of both positive linear^7^ (ie, confidence increasing in parallel with competence), and inverse^8^ (ie, confidence decreasing as competence increases) relationships. Leopold *et al* examined performance of knee injection before and after a training intervention. They observed that greater confidence correlated with poorer performance prior to the intervention but this inverse relationship reversed after instruction.^8^ There is also little correlation between level of confidence and performance for non-technical skills such as clinical or written examination grades. This has been demonstrated both in studies where the term confidence was not explicitly defined to students^9^ and where it was.^10^ Definitions are important, but confidence and competence are often simultaneously measured without clarity in their precise meaning. For example a questionnaire purporting to measure the competence levels of family residents over a 2-year period in fact asked a question on confidence rather than competence.^11^ The International Competency-based Medical Education collaboration has emphasised the importance of using precise descriptive qualifiers in definitions of competence.^12^ Despite this, there has been little examination of how healthcare professionals understand and use the terms.^13^ If confidence and competence are not clearly understood by participants in interventions, it cannot be assumed that self-reported outcomes from the learning are valid, that is, if the participants’ interpretation of confidence differs from the researchers’, how are ‘gains’ in confidence to be interpreted? In order to demonstrate the benefit of educational interventions via self-reported outcome measures, understanding how junior doctors perceive improvement in confidence and competence is therefore essential. Moreover, safe clinical practice depends on being able to recognise the limits of one's competence, so that a practitioner does not take risks, but is also not so under-confident that she/he is unable to act to prevent critical incidents. From a patient safety perspective, therefore, the relationship between confidence and competence is arguably just as important as the knowledge a clinician possesses. Understanding how doctors interpret the language used to describe their own competencies is therefore critical to patient safety interventions that seek to improve practitioners’ skills and practice.

The aim of this work was to explore junior doctors’ understanding of how they perceive their own performance, in relation to confidence and competence, in respect of managing feverish children in an emergency department. This particular subsection of patients was chosen as it is important patient safety issue in clinical practice for which clear guidance exists.^14^

## Methods

Junior doctors included in the study were in their second foundation (postgraduate) year on an Academic Foundation programme during which time is split between clinical shifts in the hospital and teaching sessions as anatomy demonstrators at the university. All of these doctors (n=14) were asked to participate in a meta-planning workshop; a modified form of focus group. There were no financial incentives to attend but lunch was provided for all participants.

This meta-planning approach was used to encourage group participation and reduce the risk, at least at the outset, of individuals not feeling confident enough to contribute.^12^ In summary the meta-planning approach differs from conventional focus groups, and indeed most qualitative approaches, in that the synthesis and analysis of themes is coproduced with the participants. Matheson and Matheson^15^ have described the process and use the technique actively in educational workshops. The group was asked a series of questions with a range of potential answers. The participants wrote their answers on individual ‘post-it notes’. All responses were then brought together and individuals were asked to highlight which answers, whether their own or others’, were their favourites using a predetermined number of votes (ie, they may use more than one vote). The selection of a favourite answer was left entirely at the discretion of the individual with no specific guidance given as to criteria for inclusion. The selection of an individual's own answer was allowed and this was stated to the participants. The highlighted responses were then discussed by the participants and overarching themes developed to group together those that were closely aligned. A discussion about these themes and other factors then proceeded.

Interested individuals were given an information sheet prior to the focus group with consent taken on the day itself. Sessions were recorded on video to enable a recording of the post-it notes and themes placed on wipe boards. Ethical approval was granted by both Leicester University and a National Health Service (NHS) regional ethics committee to undertake the meta-planning exercise.

## Results

Three focus groups took place. The first two included trainees from the foundation year commencing August 2010 and completing July 2011 (2011 Group). These meetings took place on the 12 May 2011 (group one) and 16 May 2011 (group two). Nine participants took part in 2011 group one and seven participants in 2011 group two (two of these participated in both groups). The second 2011 focus group contained a different set of questions (see online supplementary appendix 1) from the first and expanded on a theme specifically around management of the febrile child. The third workshop was for doctors in the clinical year commencing August 2011 to July 2012 (2012 Group) and took place on the 30 July 2012. Eight participants took part in the 2012 group which contained elements of both the first and second 2011 sessions.

**Figure 1:**
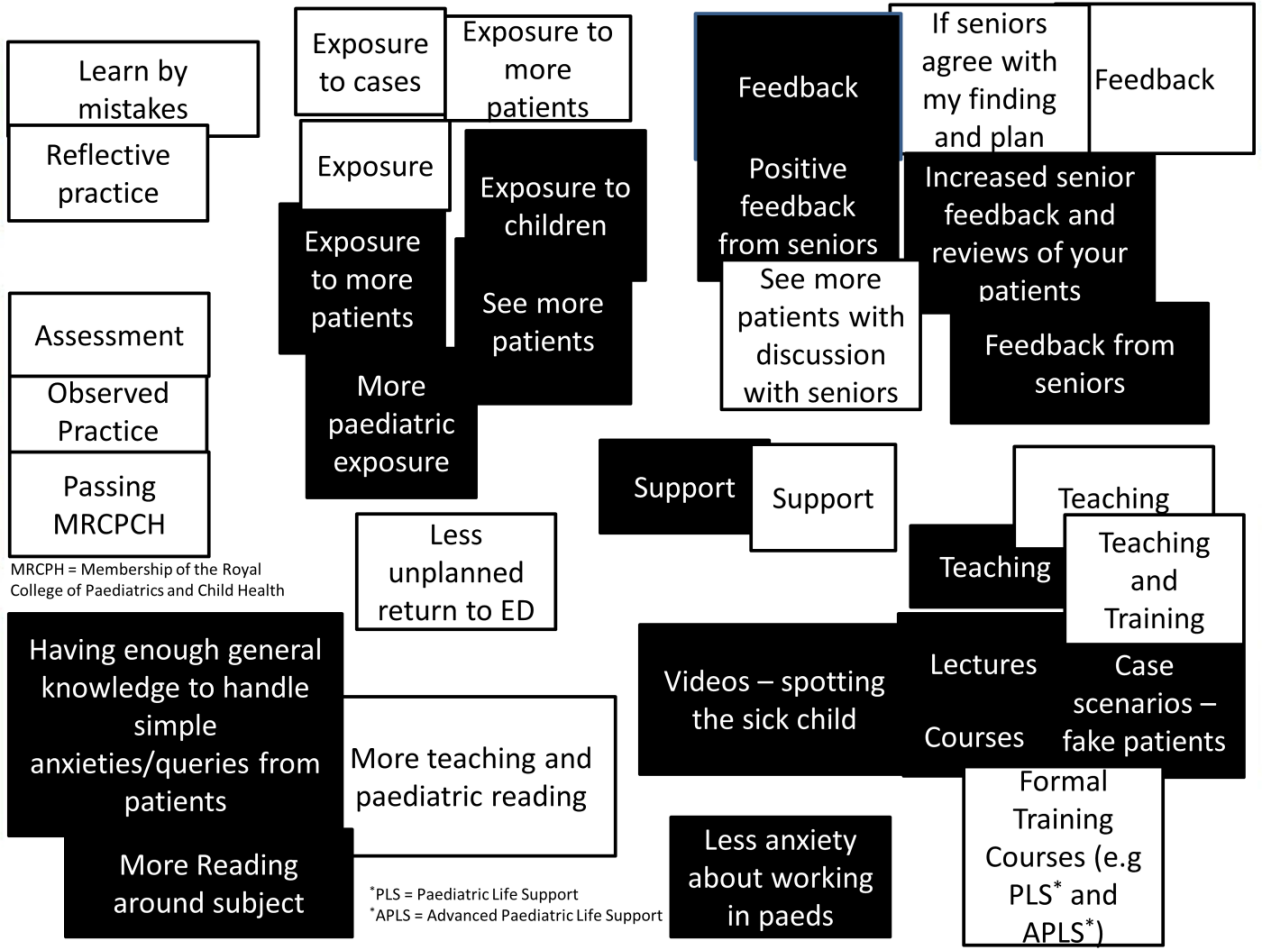
Response to question *How can I become more confident in dealing with children?* (Black Squares) and *How can I become more competent in dealing with children?* (White Squares). Groupings were performed by the participants. ED, emergency department.

A selection of responses to questions “What makes a good and bad doctor and what makes you feel confident and competent?” are shown in table 1 and to the question “What makes you feel more confident and competent?” in table 2. The questions were asked in separate parts to avoid any confusion (the complete set of responses can be found in online supplementary appendices 2 and 3). Figure 1 illustrates the meta-planning exercise collating responses to the question “what makes you feel more confident or competent in dealing with children?” The overlap between terminology used for both confidence and competence was considerable and the participants were unable to reach agreement on how to subdivide any themes that developed.

**Table 1:** Collation of themes from meta-planning exercise (what makes good and bad doctor?)

	2011		2012	
Question	Example responses	Themes	Example responses	Themes
What makes a good doctor?	Communication skills approachable good knowledge and recall Accuracy resilience problem solving skills Kind, integrity, intelligence continuous learning Decision making analytical skills	Problem solving skills Approachable Knowledgeable Resilience Competence integrity (professionalism highlighted as overarching key word)	Know own limitations Good communication skills Non-technical skills Approachability Good knowledge Team worker Caring Patient Leadership Well rounded Professionalism	Team worker Know own limitations Caring Non-technical skills Good knowledge Communication Approachability
What makes a bad doctor?	Egotistical, Does not learn from mistakes Does not listen to patients Over confident Lack of confidence Lack of knowledge Dishonesty	Arrogance Does not listen to Patients Lazy Lack of confidence Does not learn from mistakes (unsafe highlighted as overarching key word)	Poor listener Over confident/cocky Poor knowledge Over confident Poor knowledge Lone ranger Poor decision-making Poor patient Communication skills	Poor knowledge Lone ranger Poor decision Making Over confident Poor communication skills

**Table 2:** Collation of themes from meta-planning exercise (what makes you feel more confident/competent?)

	2011		2012	
Question	Example responses	Themes	Example responses	Themes
What makes you feel more competent?	Ability to perform tasks independently Completing task several/multiple times under supervision and do it correctly Feedback from senior staff Able to perform task to required standard Safe	*The 2011 groups were unable to group the competence and confidence responses under distinct headings*	Being able to perform a task and get the expected results (eg, ring blocks) Doing something correctly and appropriately To be able to achieve desired result Being left to your own devices by seniors Positive feedback Feedback from Colleagues Having done something many times before	Doing something correctly Positive Feedback Passed objective training Repeated many times Being left alone by seniors
What makes you feel more confident?	When you can go through the process independently Successful outcome on repeated occasions Gut feeling Comfortable being asked to do the task Able to teach others and give feedback You *feel* confident Experience (have done it before) When you can teach others accurately		No apprehension before carrying out procedure To approach and manage a situation with success and repetitive success Observation Experience (clinical and theoretical Teaching it and revising it When I have seen it before and spoken to someone about it/ got feedback	Repeated success Reflection positive feedback Being aware of pitfalls

### Themes from discussion

The transcripts of the discussions which resulted from the meta-planning exercise were analysed. An interpretivist approach was taken and examples of some of the comments regarding terminologies and understanding are shown below. The facilitator is (FAC) in the quotes. The year and group is stated at the bottom of each dialogue sequence.

#### Defining confidence

The groups had difficulties in clearly defining and distinguishing between the terms confidence and competence, although they did recognise a difference. For ‘confidence’ in particular, the importance of recognising over and under-confidence was mentioned by both 2011 groups and 2012 group. Encapsulating when someone became overconfident or under-confident was highlighted as challenging:

(7)—It could also be about lack of confidence. What is the boundary for lack of confidence and overconfidence? [It] Can sometimes be if you lack confidence then you might want to double-check your plan or double-check your management with someone more senior to make sure you're safe, as ultimately it's about the patient's safety than you thinking “yes I have the right diagnosis” [2011 Group One]

#### Defining competence

As with confidence, the groups could not develop an agreed definition of competence. Participants were surprised by the difficult they had in reaching a clear understanding of its use:

(FAC)—Ok, you have started to allude to it already, what I want you to do is the same. “How do you know when you are competent at something?”

(6)—What is the proper word that we mean? Because when we first started, if you were competent at something you were good and you were safe and you could do it well. When we started here we got taught that competence means that you could do it to the bare minimum expected standard without actually doing any harm, which is not what it meant to me? [2011 Group One]

Although the groups found defining the nature of competence difficult, its importance was clear to them. One candidate commented that they had certain competencies, but not at a high enough level to make some key clinical decisions. Increasing exposure, and in their terms ‘pattern recognition’, were one way competency could be reached:

(2)—Fear of the unknown and fear of whether you are competent. So at the beginning, and also it's a bit of pattern recognition. A good example is—I didn't know how sick someone had to be to qualify for going into resus, so it's not that I didn't recognise the patient was sick; I just didn't know at what point they were sick enough to go to resus. That's a pattern recognition thing, so you start to know, OK I want to admit my patient to resus now, and it comes with experience. Whereas at the beginning there were multiple times where you just didn't really know what happens. [2011 Group one]

#### Competence versus confidence

Despite not being able to completely allocate components of the two terms to themes, as demonstrated in figure 1, individual participants from both year groups could identify a difference between them.

(8)—So if you are competent at something, it means you are able to do that procedure at the required level. If you are confident it means you are happy with your skills and you can go on and do it. So you could be competent but you might not be confident, as it depends on what the standard of competences based on, so you are confident with your own standards you need to acquire. [2011 Group One]

(6)—I'd say that confidence is subjective, to do a task, whereas competence would be more of an objective measure of “are they actually able to do that job?” [2011 Group Two]

Evident in these kinds of comments was the interaction between competence and confidence. At a theoretical level, they could be distinguished from each other and defined completely; but at a practical level, both were crucial to being a proficient professional with the requisite skills and personal attributes to contribute to the effective care of patients. As this realisation emerged through the course of the discussions, participants in the groups began to suggest alternative, integrated concepts that made more sense to them as a means of assessing their own performance by bringing together the most important aspects of competence and confidence.

#### Alternatives to using confidence and competence

Both groups felt that there were other approaches that could be used in place of confidence and competence as standard terms. The junior doctors themselves picked up on each other's use of language:

(7) So for me it's a change in practice rather than me saying I feel more confident if you have a change of practice for the better.

(3)—Also I think getting away from those words. Actually what (7) was talking about is competence, she is just putting it in her own words and if people don't understand the difference between competent and confidence what you do is use words where you say to them—do you feel that you have learnt more about this procedure and do you think it will affect your practice? [2011 Group one]

One term repeatedly mentioned was ‘safe’, which was commonly understood and agreed universally by the 2011 group as a unifying term:

(3) I think unsafe is almost an umbrella term, kind of like professionalism, because if you are overconfident or if you are under-confident, if you are lazy then any of those things then you are unsafe [2011 Group One]

(FAC) Is there a term that you think you use either instead of confident or competence or is there one that you use to describe both?

(6) Experience

(5) I feel safe as well

(4) I think so

(5) So that the department itself feels safe that I am working there, but I also feel safe

Group: Yes [2011 Group Two]

(5) Also if some senior described me as being a safe doctor I think that is probably one of the biggest compliments in the A&E Department that you can have [2011 Group Two]

This concept was tested with the 2012 Focus Group and the concept appeared to have face validity for this group as well:

(FAC) If I asked you doing what you have just done in the last year. Do you feel safe managing a child with a rash and a fever who may be unwell?

Group: Yes

(FAC): Do you see a difference? If I ask a question: Do you feel confident managing a febrile child with a fever and a rash versus do you feel safe managing a child with a fever and a rash. Do you see a difference between those?

Group: Yes [2012 Group]

Doctors perceiving they lack both confidence and competence are likely, as demonstrated by the responses to the focus groups, to ask for help, as they recognise they are unable to intervene in a manner beneficial to patients. However, those who perceive themselves to be competent (assuming they are correct in this insight) but are not confident to apply them may cause harm by omission, that is, not acting in an emergency when their intervention would have been beneficial. Conversely, again assuming their perceptions are correct; those who rate themselves confident and competent are likely to provide good patient care. Finally those who are confident but potentially without the correct competencies may attempt procedures they are not proficient to perform (therefore risking patient safety). Table 3 shows a matrix of reported confidence versus competence and how this may affect patient care based on these extrapolations from the focus groups.

**Table 3:** Proposed relationships between reported competence and confidence and actual clinical performance

		Reported competence	
		Low	High
Reported confidence	Low	Clinical performance: safe	Clinical performance: suboptimal
	High	Clinical performance: unsafe	Clinical performance: safe

The junior doctors’ own comments regarding their perceptions lend support to the proposed matrix. Given the lack of reliability in defining competence and confidence it is likely the grid represents performance at the extremes rather than a tool that can be applied to all healthcare professionals.

## Discussion

The focus groups demonstrated, across two different years, that although the junior doctors recognised a difference between confidence and competence, they were not able to agree on exactly how. In fact they often grouped descriptors under competence and confidence headings (figure 1). This may help to explain why previous studies have shown no relationship between self-ratings of confidence and actual competence.^10^ Little work exists that seeks to explain this weak relationship, though one exception is Stewart *et al*'s^13^ work on junior doctors’ interpretation of these terms. In interviews with four junior doctors, of 1 year less experience than those in this work, similar themes emerged of difficulties with encapsulating exactly what the two terms meant. Stewart *et al*^13^ concluded: “Asking a house officer whether they are confident to perform a task will not identify their beliefs about their competence. Neither will asking them whether they are ‘competent’ to give information on what they would be willing to perform.”

The Stewart study was over a decade ago, but our study suggests that the difficulty with utilising competence and confidence as terms to evaluate educational interventions remain. Certainly there are current examples of unclear applications of the terms in research and training. A recent study looking at the impact of an online learning package in evidence-based medicine used a self-evaluation questionnaire^16^ based on scale which had not defined the term confident in its validation stage.^17^ Ultimately questions regarding self-assessed competence and confidence following a training intervention may be misinterpreted unless clarifying statements are used to qualify the researcher's meaning. Self-perception of learning gain following an intervention is an important part of determining educational efficacy^18^ so it is important these terms are interpreted correctly.

### Competence versus confidence

Are competence and confidence the best terms to use, even with clear definitions for their use? The appropriateness of the concept of competence in particular has come in for criticism in the literature. Competency has been challenged by Brooks,^6^ who argues that competency has arisen from a very behavioural model where the theory would imply that “training a doctor is qualitatively no different than training a touch-typist.”^6^ For Brooks, a focus on competency risks measuring only against the ability to acquire skills necessary to complete tasks, rather than assessing the broader knowledge and values needed to function as a professional. The difficulties of the junior doctors in this study arguably reflect this tension between task-focused competency and the more complex reality of becoming a proficient practitioner in a professional sphere. Others highlight the difference between the way in which experts complete tasks compared to beginners.^19^ ^20^ This implies components of ‘competency’ may alter as an individual becomes more skilled, and perhaps more confident.

An outcome of the focus group work was a potential additional self-assessment question in respect of evaluation. The utilisation of perceived safety as a self-assessment measure has a potentially important practical application in healthcare. Although the junior doctors had difficulty defining competence and confidence, there was general consensus on what it was to feel ‘safe’ in the management of the febrile child. The concept of patient safety has been previously defined as “the prevention of harm to patients”,^21^ but the utilisation of ‘safeness’ as an evaluative term has only been explored in the patient safety literature. Even in this literature the evaluation centred on the teaching of patient safety^22^ rather than the perception of feeling safe in a particular competency domain.

Brooks^6^ argued that competency could only be a subjective process as another competent person, however ‘expert’, was required to make this judgement. He felt objective criteria would infer that competency is independent of the assessor. Creating such a grid, like the ROLMA matrix,^23^ provides a way for clinicians to understand this potentially complex educational theory and being able to act on the findings. The importance of this becomes apparent if self-evaluation of safety is added to the matrix. Even without a concrete definition the focus groups would imply that those in the low confidence, low competence category would score themselves as unsafe (table 4) which would likely to be contrast to their actual clinical performance (table 3) while those in the low competence, high-confidence category would score themselves as safe while their clinical performance was unsafe.

**Table 4:** A proposed matrix of perceptions of competence, confidence and safety

		Reported competence	
		Low	High
Reported confidence	Low	Self-perception: unsafe	Self-perception: unsafe or Safe
	High	Self-perception: safe	Self-perception: safe

Little research has been performed on junior doctors’ assessment of their own safety. In a qualitative study examining the causes of prescribing errors, junior doctors noted lack of personal knowledge and experience as key reasons for their mistakes^24^ but a prior assessment of their perception of competence and confidence was not made. The recording of perception of safeness may be used with the confidence and competence assessment to highlight individuals most in need of an intervention or requiring support. For example those individuals ranking low on competence but high on confidence perceiving themselves as safe (as per table 4) may require additional supervision in the workplace. This adds a visual representation to work on understanding the overestimation of performance by individuals of low competency levels.^25^ These individuals are at particular risk because they have little or no insight into their weaknesses. Triangulation using the matrix in tables 3 and 4 may be helpful to others interpreting these subjective perceptions.

Other tools which enable individuals to examine their own beliefs may be used in parallel with this process to gain further insights into behaviours. The Johari window,^26^ a methodology where participants select characteristics which they think best reflect them, and these are compared with their peers’ views of the characteristics that best reflect them, would be one such approach. Furthermore if complemented with results from a 360° appraisal, then supervisors may be able to gain insights into the validity of their juniors’ self-perceptions. However, this perception of safeness is not an overall indicator of patient safety in that patient outcomes are not being examined directly. However, it may be possible to match perception of safety with actual clinical performance over the period of doctor's attachment. This would be a relevant area for future research.

### Limitations

There are a number of limitations with this study design in drawing the conclusions made. The groups were a specific cohort of junior doctors (chosen because of their increased availability to partake in the study) who may not be representative of all doctors on the foundation programme. Those on the academic programme may have different motivations or perceptions and have all previously shown an aptitude for teaching or research. Some of the thinking of the junior doctors was at relative high levels in Bloom's taxonomy.^27^ They were synthesising and evaluating on their reflections over the year and this may not be representative of other groups of junior doctors who may be at lower levels of the taxonomy.

Importantly this work was only performed on groups of junior doctors. Other healthcare professionals may not have similar views on the difficulties in interpretation of confidence and competence. This may be particularly relevant for a consultant group who may have either increased insight into their own abilities or perhaps less insight through the habituation of the frequent performance of skills and application of knowledge. It also did not include the viewpoints of patients who may have different perceptions on the doctor's ability. Furthermore ‘safeness’ in the context of the patient's clinical management may not be applicable in other situations such as patient counselling or leading an arrest for example. Culture and environment are important to outcomes of learning^28^ and it is possible that the particular circumstances within the emergency department, even over a 2-year cycle, were unique and cannot be replicated elsewhere. Testing the external validity of both these findings and the conceptual framework itself are therefore important.

The themes from the meta-planning phase of the focus groups were extremely useful in formulating the presented conclusions; however they were quite time consuming for the participants. Modifications to the workshops in 2012 aimed to mitigate this but further discussion, and perhaps, individual interviews may have given more detailed information conflicting with some of the theories drawn. Future research may wish to examine via focused interviews the proposed constructs, not just with junior doctors, but also with medical educators and consultants.

## Conclusion

Competency and Confidence are confusing terms for junior doctors but reported ‘safeness’ may help healthcare professionals in training roles explore underlying performance concerns. The tables, based on junior doctors’ own experiences, will hopefully help promote discussion around the terms competence and confidence. Used in conjunction with other forms of evaluation this could then lead to the development of a conceptual framework and greater insight on the part of the junior doctors and greater understanding on the part of those training them.
